# Clinical outcomes of pericapsular nerve group block in hip arthroscopy: A systematic review and meta‐analysis

**DOI:** 10.1002/jeo2.70303

**Published:** 2025-07-24

**Authors:** Jeroen den Breejen, Justin van Loon, Inger N. Sierevelt, Michel H. Klaver, Daniel Haverkamp

**Affiliations:** ^1^ Xpert Clinics Orthopedie Amsterdam the Netherlands; ^2^ Department of Orthopedic Surgery, Amsterdam University Medical Centers, Academic Medical Center, Amsterdam Movement Sciences University of Amsterdam Amsterdam the Netherlands; ^3^ Department of Orthopedic Surgery Spaarne Gasthuis Academy Hoofddorp the Netherlands; ^4^ Department of Anesthesiology Zaandam Medical Center Zaandam the Netherlands

**Keywords:** hip arthroscopy, PENG block, pain management, regional anaesthesia

## Abstract

**Purpose:**

In hip arthroscopy, regional anaesthesia techniques are gaining importance for pain management. A side effect of nerve blocks can be unintended motor block, affecting early post‐operative mobilization. Pericapsular nerve group (PENG) block is a technique that may address this issue. This review compares the clinical effects of the PENG block with current pain management practices in hip arthroscopy.

**Methods:**

A literature search was conducted in PubMed, Embase and the Cochrane Library, following the PRISMA guidelines. The primary outcome was post‐operative pain. Secondary outcomes included duration of hospital stay, opioid use, post‐operative motor function and complications. Outcomes were presented in subgroups categorized by study design (randomized controlled trials and retrospective comparative cohort studies). Risk of bias was assessed with both the Cochrane checklist and MINORS. Quality of evidence was evaluated using the Grades of Recommendation Assessment, Development and Evaluation.

**Results:**

This systematic review included four RCTs and six retrospective comparative cohort studies, comprising 900 patients in total. The RCTs reported lower mean pain scores at 24 h post‐operatively and lower maximum pain scores at the post anaesthesia care unit (PACU) in the PENG group, with no differences observed in PACU time, opioid usage at the PACU or post‐operative nausea and vomiting (PONV). The retrospective cohort studies revealed lower maximum and mean PACU pain scores, reduced PACU time, decreased opioid usage and a lower incidence of PONV in the PENG group. One RCT reported better quadriceps strength in PENG, whereas one RCT and cohort study reported no motor impairment in this group.

**Conclusions:**

PENG block holds promise for post‐operative pain relief, decreased opioid consumption, shorter PACU stay and potentially less PONV without affecting motor function. No differences in hospital stay or PACU opioid use were found, necessitating further research. Due to minimal risks and potential advantages, PENG could be a viable option for analgesia in hip arthroscopy.

**Level of Evidence:**

Level III, review including Level I–III studies.

**Abbreviations**
BKK: bupivacaine, ketamine and ketorolac
BMI: body mass index
CI: confidence interval
FICB: fascia iliaca compartment block
FN: femoral nerve
GA: general anaesthesia
GRADE: Grades of Recommendation Assessment, Development and Evaluation
IQR: interquartile range
LFCN: lateral femoral cutaneous nerve
MME: milligram morphine equivalent
NRS: numerical rating scale
PACU: post anaesthesia care unit
PENG: pericapsular nerve group
PI: pericapsular injection
PONV: post‐operative nausea and vomiting
RCT: randomized controlled trial
SD: standard deviations
VAS: visual analogue scale

## INTRODUCTION

In the last 15 years, hip arthroscopy has gained prominence in the field of orthopaedics [5]. Compared with open surgery, arthroscopic surgery is recognized to be minimally invasive, preserving surrounding tissues from unnecessary trauma and exposure [4]. It facilitates the diagnosis and treatment of many hip pathologies with the advantages of less morbidity and faster recovery times. Despite these advantages, post‐operative pain remains an important concern for patients and healthcare practitioners [39]. Effective analgesia is necessary to relieve patient discomfort, facilitate early mobilization, reduce hospital stay and increase overall patient satisfaction.

Regional anaesthesia techniques have gained importance in recent years in pain management strategies in orthopaedic surgery. The blocks commonly used in hip arthroscopy are the femoral nerve block (FNB) and the fascia iliaca compartment block (FICB) [39]. An important side effect can be unintended motor block, resulting in weakness of the quadriceps muscles, which impairs early post‐operative mobilization [10, 11]. One of the recently developed techniques to address this problem is the pericapsular nerve group (PENG) block, which was first described by Girón‐Arango et al. [12]. It has attracted attention for its potential to provide efficient post‐operative pain relief without impairing motor function. This is achieved because the PENG block exclusively affects the articular branches of the F, obturator nerve and accessory obturator nerve without affecting the motor branches of the FN responsible for innervating the quadriceps muscles, which is not achieved by other blocks [16, 17]. As a result of its potential for pain relief, PENG block is hypothesized to also reduce intra‐ and post‐operative opioid usage and hospital stay.

The literature investigating the potential effects and safety of PENG block is limited, with most studies focusing on other topics, such as hip fracture and arthroplasty. To our knowledge, only one review has been conducted on the clinical effects of PENG block in hip arthroscopy in April 2024, investigating fewer outcomes and studies compared to our study [33]. Our review aims to describe a broad range of outcomes to fully evaluate the effect of PENG block. Therefore, we also included motor function, adverse events and post‐operative nausea and vomiting (PONV). To provide a better and more complete overview of PENG block, we included additional studies and performed a meta‐analysis. The aim of this review is to investigate the clinical effects of PENG block in comparison with currently available practices for pain management in hip arthroscopy patients, with a focus on post‐operative motor function, post‐operative pain, opioid consumption, duration of hospital stay, complications and side effects.

## METHODS

### Search methods

This systematic review was preregistered in PROSPERO (registration number: CRD42023472769) and performed following the PRISMA 2020 statement: an updated guideline for reporting systematic reviews [26]. For this systematic review, a literature search was conducted in the PubMed, EMBASE and Cochrane Library databases (up to 15 December 2024) to examine the effectiveness of PENG block in patients who underwent hip arthroscopy. The search was built with the aid of a clinical librarian, and the complete search strategies are shown in Appendix A.

### Screening and selection of studies

The abstract and full‐text screening were performed separately by two reviewers (JL and JB). Disagreements on the selection of studies were resolved through discussion and consultation with a third reviewer (MK). Studies were included when patients underwent hip arthroscopy and when PENG block was used as an analgesic procedure. Studies were excluded if they did not focus on hip arthroscopy or involved additional procedures such as periacetabular osteotomies, involved alternative analgesic methods, had noncomparative designs or were a study protocol. A heterogeneous comparison group was chosen because of the limited available literature, comprising different analgesic methods and various indications for hip arthroscopy for various indications. All comparative studies, including RCTs and retrospective comparative cohort studies, were included and analyzed in subgroups to provide the best overview. Covidence was used for the screening and selection of the articles [18].

### Data extraction

Data on the study design, year of publication, sex, age and indication for hip arthroscopy were extracted. For the intervention, the type of infiltration anaesthetic and concentration used for the PENG block were collected. The same data were collected for all reference blocks (sham, FICB and no block).

### Outcomes

The primary outcome assessed in this study was post‐operative pain, which was measured via the visual analogue scale (VAS) and the numerical rating scale (NRS). Post‐operative pain scores were documented directly post‐operatively at the post anaesthesia care unit (PACU) up to 24 hours following arthroscopy. The secondary outcomes were post‐operative motor weakness, opioid consumption up to two weeks post‐surgery, the duration of hospital stay and PACU admission, the occurrence of complications and PONV. Opioid consumption was recorded as the milligram morphine equivalent (MME).

### Risk of bias

Critical appraisal was conducted independently by two reviewers (JL and JB) via the Cochrane Collaboration's tool for RCTs and the MINORS criteria for both the RCTs and retrospective cohort studies [15, 32]. MINORS scores were also reported for RCTs to compare bias across all included studies. The Cochrane tool assesses 12 domains, including random sequence generation, allocation concealment, blinding and attrition bias, with studies rated as having high (red), unclear (orange) or low (green) risk of bias. The MINORS criteria evaluate 12 items, scoring studies as ‘not reported’ (0 points), ‘inadequate’ (1 point) or ‘adequate’ (2 points), with a maximum score of 24.

### Qualitative analysis

The quality of evidence and the strength of the outcomes were evaluated by two reviewers (JL and JB) using the Grades of Recommendation Assessment, Development and Evaluation (GRADE) method [14]. This tool assigns grades to outcomes on the basis of the strength of their evidence, categorizing them into four levels of strength, which range from ‘very low’ to ‘strong’.

### Statistical analysis

The study, along with additional patient characteristics such as age and sex and clinical practices such as the intervention, comparison and surgical procedures, are reported through descriptive statistics. Means with standard deviations (SDs) are calculated for continuous variables, and categorical variables are presented in numbers with accompanying proportions. We also documented the sex and percentage of men in each study group. Primary outcomes were the mean pain scores in the PACU, the mean maximum scores in the PACU and the mean pain scores 24 h post‐operatively. The mean scores with standard deviation were documented. In case at least three studies recorded the same outcomes and used identical analgesic methods for each group, data were pooled and mean differences with 95% confidence intervals (CIs) were calculated and pooled based on a random effect model with inverse variance weighting. If the 95% CI did not contain the value 0, mean differences were considered statistically significant. Outcomes were categorized and pooled in subgroups according to the study design. Heterogeneity was determined using the *I* ^2^ statistic, with cut‐off values of 50% indicating significant heterogeneity [13]. Statistical analyses were performed with R version 4.0.4 (R Foundation for Statistical Computing) using a meta package for meta‐analyses [2]. Statistical analysis was performed by a statistical expert (IS).

## RESULTS

### Search results

**Figure 1:**
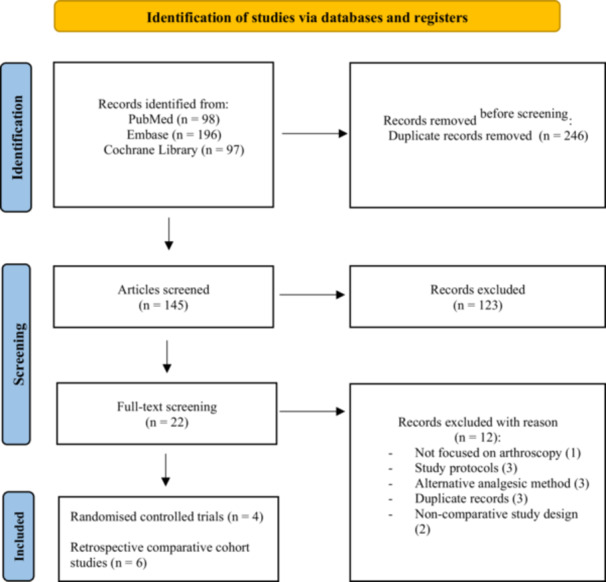
PRISMA flow diagram.

A search was performed in PubMed, Embase and the Cochrane Library on 15 December 2024, identifying 98, 196 and 97 studies, respectively. Of those studies, 246 were duplicates. The titles and abstracts of the 145 remaining studies were screened. Among these studies, 123 were excluded because the PENG block was not specifically examined or because the PENG block was used for procedures other than hip arthroscopy. Among the 22 remaining studies, 12 were excluded after full‐text evaluation. Consequently, 10 studies with a total of 900 patients were included in this systematic review (Figure 1) [1, 6, 9, 19, 20, 21, 24, 27, 35, 38].

### Study characteristics

This review included 10 studies, 4 randomized controlled trials (RCTs) and 6 retrospective comparative cohort studies. All of these studies were published from 2022 to 2024. The RCTs included 257 patients in the analysis, of whom 130 received a PENG block. Among all patients, 57% were male, the mean age was 33 years, and the mean body mass index (BMI) was 25.1. The retrospective comparative cohort studies involved a total of 643 patients. Four studies reported the mean age of the participants, with an overall mean of 30 years. Two studies reported only the median age and median BMI, whereas Widmeyer et al. did not record the percentage of males or BMI data [6, 19, 35]. Among the remaining five retrospective comparative cohort studies, male participants accounted for 47% on average. A detailed summary of all the baseline characteristics is presented in Table 1.

**Table 1:** Study characteristics.

Study	Groups	Patients *n*	Male *n* (%)	Age mean (SD)	BMI mean (SD)	Procedures
**Randomized controlled trials**						
Liu et al. [21]	PENG/LFCN FICB	39 39	22 (56) 24 (62)	44 (14) 39 (18)	25 (4) 24 (4)	—
[24]	PENG FICB	23 20	11 (48) 14 (70)	29 (6) 27 (6)	25 (5)	Cam repair debridement Labral repair Loose body removal Pincer repair
[9]	PENG Sham block	34 34	23 (68) 24 (71)	31 (6) 30 (7)	25 (2) 24 (3)	Femoroplasty Acetabuloplasty Debridement Labral repair Chondroplasty
[1]	PENG Sham block	34 34	15 (44) 14 (41)	33 (10) 29 (10)	27 (5) 26 (5)	Femoroplasty Acetabuloplasty Labral repair Capsular plication
**Retrospective comparative cohort studies**						
[6]^a^	PENG QL	50 50	22 (44) 13 (26)	36 [28, 42] 33 [27, 39]	29 [25, 37] 29 [24, 33]	Femoroplasty Labral repair
[19]^a^	PENG/LFCN Neuraxial PENG Neuraxial	86 86 26 26	37 (43) 37 (43) 17 (65) 17 (65)	34 [24, 45] 34 [24, 45] 31 [23, 44] 31 [23, 44]	24 [22, 28] 24 [21, 26] 23 [21, 26] 25 [23, 32]	—
[35]	PENG/BKK GA GA/Marcaine	20 20 12	—	33 (11) 27 (10) 30 (10)	—	Labral repair debridement Femoroplasty acetabuloplasty
[38]	PENG GA	28 25	10 (36) 10 (40)	36 (14) 31 (10)	25 (4) 25 (3)	Labral repair Labral reconstruction Femoroplasty Acetabuloplasty
[20]	PENG GA	25 25	12 (48) 11 (44)	27 (10) 26 (9)	26 (5) 26 (4)	Femoral neck osteoplasty Acetabuloplasty Labral repair Capsularclosure
[27]	PENG GA	75 89	43 (57) 43 (48)	34 (12) 30 (11)	26 (4) 25 (4)	Labral repair Osteoplasty Labral debridement Capsulotomy Cam lesion repair or resection Pincer lesion repair or resection Psoas release

Abbreviations: BKK, bupivacaine, ketamine and ketorolac; BMI, body mass index; GA, general anaesthesia; FICB, fascia iliaca compartment block; IQR, interquartile range; LFCN, lateral femoral cutaneous nerve; PENG, pericapsular nerve group; SD, standard deviation.

^a^ These studies used median scores [IQR] for age and BMI.

All patients underwent hip arthroscopy for various arthroscopic surgical procedures, as shown in Table 2. In eight studies, PENG block was the sole local analgesic administered. One RCT and a retrospective comparative cohort study combined PENG block with lateral femoral cutaneous nerve (LFCN) block, whereas another retrospective comparative cohort study combined PENG block with a pericapsular injection (PI) containing bupivacaine, ketamine and ketorolac (BKK) [19, 21, 35]. The control groups in the studies received different anaesthetic interventions. Two RCTs compared the PENG block to FICB, and the other two used a sham block (0.9% saline) [1, 9, 21, 24]. Four retrospective comparative cohort studies compared PENG block with general anaesthesia (GA) alone [19, 20, 27, 35, 38]. Widmeyer et al. also included a third group that received GA along with a PI of Marcaine. The other two retrospective comparative cohort studies used a quadratus lumborum (QL) block and neuraxial analgesia in their control groups [6, 19]. The types and concentrations of analgesics used in each group are documented in Table 2.

**Table 2:** Types of analgesia per group.

Study	Groups	Analgesia
**Randomized controlled trials**		
[21]	PENG/LFCN FICB	35 mL 0.375% ropivacaine/35 mL 0.375% ropivacaine + GA 40 mL 0.375% ropivacaine + GA
[24]	PENG FICB	20 mL 0.5% bupivacaine + GA 20 mL 0.5% bupivacaine + GA
[9]	PENG Sham block	20 mL 0.375% ropivacaine + GA 20 mL 0.9% saline + GA
[1]	PENG Sham block	20 mL 0.5% ropivacaine + GA 5 mL 0.5% saline + GA
**Retrospective comparative cohort studies**		
[6]	PENG QL	20 mL 0.35%–0.5% ropivacaine 30 mL 0.35% ropivacaine
[19]	PENG/LFCN Neuraxial PENG Neuraxial	15–20 mL 0.25% bupivacaine/5–10 mL 0.25% bupivacaine Spinal or combined spinal epidural 15–20 mL 0.25% bupivacaine Spinal or combined spinal epidural
[35]	PENG/BKK GA GA/Marcaine	0.5% ropivacaine/50 mL bupivacaine, ketamine, ketotolac + GA GA 20 mL marcaine + GA
[38]	PENG GA	15–20 mL bupivacaine or ropivacaine (patient dependent) + GA GA
[20]	PENG GA	20 mL ropivacaine + GA GA
[27]	PENG GA	10–20 mL 0.5% bupivacaine or ropivacaine + GA GA

Abbreviations: BKK, bupivacaine, ketamine and ketorolac; GA, general anaesthesia; FICB, fascia iliaca compartment block; LFCN, lateral femoral cutaneous nerve; PENG, pericapsular nerve group.

### Risk of bias

The results of the risk of bias assessment, which focused on the primary outcome, are detailed in Appendices B and C. The four RCTs demonstrated an overall low risk of bias. Additionally, one RCT lacked the statistical power to fully support its findings [21]. For retrospective comparative cohort studies, the main sources of bias were the absence of (or failure to report on) blinding and the lack of a sample size calculation.

### Qualitative analysis

The results of the GRADE assessment are shown in Appendix D. The strength of evidence for primary outcomes varied across study types. In RCTs, the evidence for the mean VAS score in the PACU and 24 h post‐operatively was moderate, despite their moderate risk of bias. For the maximum VAS score in the PACU, the strength of evidence was high. In contrast, retrospective comparative cohort studies showed very low strength of evidence for both the maximum and mean VAS scores in the PACU, primarily due to a high risk of bias.

For the secondary outcomes of the RCTs, the evidence was moderate for time spent in the PACU, opioid use in the PACU and PONV. However, evidence for opioid use 24 h post‐operatively was low due to inconsistent results and minor bias, whereas evidence for the timing of the first analgesia request was high. In retrospective comparative cohort studies, the strength of evidence for time spent in the PACU, opioid use (both in the PACU and intraoperatively), time to discharge, and PONV was very low, largely owing to a very high risk of bias. The results for each outcome are presented in Tables 3 and 4.

### Primary outcome: Post‐operative pain

Data on maximum PACU VAS scores, mean PACU VAS scores and mean 24‐h post‐operative VAS scores are presented in Table 3.

**Table 3:** Primary outcome.

Study	Groups	Max VAS PACU score (SD)	*p* **value**	GRADE	Mean VAS PACU score (SD)	*p* **value**	GRADE	Mean VAS 24 h post‐operative score (SD)	*p* **value**	GRADE
**Randomized controlled trials**										
[21]	PENG/LFCN FICB			High			Moderate	1 [1, 2]^a^ 3 [2, 4]^a^	<0.001	Moderate
[24]	PENG FICB	6^a^ 8^a^	<0.001		3^a^ 6^a^	<0.001		2^a^ 3^a^	<0.001	
[9]	PENG Sham block				4.5 (1.8) 4.6 (2.3)	0.861		1.3 (0.9) 2.4 (1.6)	0.009	
[1]	PENG Sham block				6.1 (2.6)^b^ 6.9 (2.2)^b^	0.17		4 (2.4)^b^ 4 (1.8)^b^	0.98	
**Retrospective comparative cohort studies**										
[6]	PENG QL	6 [4, 8]^a^ 7 [5, 9]^a^	0.4098	Very low			Very low			
[19]	PENG/LFCN Neuraxial PENG Neuraxial	6.0 (2.4)^b^ 6.8 (2.1)^b^ 6.3 (2.4)^b^ 6.4 (2.2)^b^	0.01 0.837		3.9 (2.1)^b^ 3.4 (1.7)^b^ 3.8 (2.1)^b^ 3.0 (1.0)^b^	0.095 0.138				
[35]	PENG/BKK GA GA/Marcaine				3.9 (3.7) 7.7 (2.1) 6.6 (2.3)	<0.001 0.048				
[38]	PENG GA	5.3 (2.1) 7.0 (1.9)	0.004							
[20]	PENG GA	6.5 (2) 7.4 (1.6)	0.08							
[27]	PENG GA	5.5 (2.5) 6.5 (2.5)	0.02		3.5 (2.0) 4.2 (2.0)	0.03				

Abbreviations: BKK, bupivacaine, ketamine and ketorolac; GA, general anaesthesia; GRADE, Grades of Recommendation Assessment, Development and Evaluation; FICB, fascia iliaca compartment block; IQR, interquartile range; LFCN, lateral femoral cutaneous nerve; NRS, numerical rating scale; PENG, pericapsular nerve group; SD, standard deviation.

^a^ These indicate median [IQR] scores, not mean scores.

^b^ NRS score instead of the VAS score.

#### Mean VAS scores in PACU

Three RCTs found no significant difference in mean pain score in PENG compared to sham and a lower median VAS score with PENG compared to FICB, while two retrospective comparative cohort studies found significantly lower mean VAS scores in the PACU in the PENG group compared to the GA group [1, 9, 24, 27, 35].

#### Mean VAS scores 24 h post‐operatively

Three RCTs found significantly lower 24‐h post‐operative median and mean VAS scores in the PENG group compared to the FICB and sham groups [9, 21, 24]. The study by Liu et al. also reported lower median pain scores at 6, 12, 18, 36 and 48 h post‐operatively in the group that received a combination of PENG and LFCN (scores: 3, 3, 2, 1, 1, 1) compared to the group that received FICB (scores: 4, 3, 3, 3, 3, 2) [21].

#### Maximum VAS score in PACU

**Figure 2:**
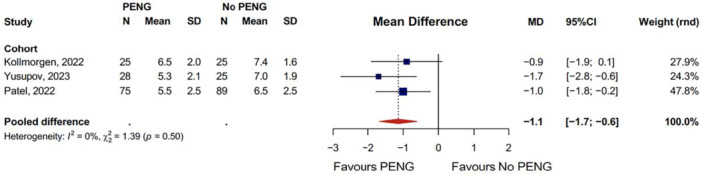
Pooled maximum VAS scores in the PACU. CI, confidence interval; MD, mean difference; PACU, post anaesthesia care unit; PENG, pericapsular nerve group; SD, standard deviation; VAS, visual analogue scale.

In the RCT group, one study found that the median maximum VAS scores of patients in the PACU are significantly lower in the PENG group [24]. One retrospective comparative cohort found significantly lower maximum NRS pain scores in the group that combined PENG with LFCN block compared to the group that received no block [19]. The other three retrospective comparative cohort studies were pooled in a meta‐analysis for the maximum VAS scores in the PACU (Figure 2). These studies showed a significantly lower max VAS in the PACU with PENG compared to no PENG (mean difference [MD]: −1.1, 95% CI: [−1.7 to −0.6]) [20, 27, 38].

### Secondary outcomes

Data on intraoperative opioid use, total time spent in the PACU, total opioid consumption in the PACU, total opioid use 24 h post‐operatively, time to first analgesia, incidence of PONV and time to discharge are presented in Table 4.

**Table 4:** Secondary outcomes.

Study	Groups	Time spent in PACU min (SD)	*p* **value**	GRADE	Opioid use PACU MME (SD)	*p* **value**	GRADE	Opioid use 24 h post‐operative MME (SD)	*p* **value**	GRADE	PONV *n* (%)	*p* **value**	GRADE
**Randomized controlled trials**													
[21]	PENG/LFCN FICB			Moderate			Moderate			Low	2 (5) 2 (5)	1.000	Moderate
[24]	PENG FICB							16.5 (9.9) 27.5 (9.6)	0.05				
[9]	PENG Sham block							3.5 (1.7) 3.6 (1.8)	0.88		1 (3) 2 (6)	0.54	
[1]	PENG Sham block	143 (49.2) 142 (61.9)	0.9		7.5 (4.2) 8.7 (4.4)	0.3		10.2 (4.8) 8.6 (2.8)	0.1		3 (9) 9 (27)	0.1	
**Retrospective comparative cohort studies**													
[6]	PENG QL	72 [56, 94]^a^ 90 [77, 113]^a^	0.0008	Very low	15 [8, 30]^a^ 15 [8, 33]^a^	0.9611	Very low						Very low
[19]	PENG/LFCN Neuraxial	144 [108, 204]^a^ 246 [210, 294]^a^	<0.001		14.6 (5.8) 26.0 (13.0)	<0.001					10 (12) 15 (17)	0.279	
	PENG Neuraxial	204 [162, 258]^a^ 294 [228, 342]^a^	0.0023		22.0 (15.8) 27.7 (15.0)	0.108					4 (15) 3 (12)	0.291	
[35]	PENG/BKK GA GA/Marcaine	47 (44) 99 (40) 129 (59)	<0.001 <0.001		4.1 (5.7) 26.3 (18.3) 28.1 (19.3)	0.001 0.001							
[38]	PENG GA	129 (34) 161 (50)	0.008		14.4 (11.1) 31.2 (20.1)	<0.001					0 (0) 4 (16)	0.043	
[20]	PENG GA	81.5 (19) 95.8 (31)	0.05		34.3 (12.1) 50.3 (11.2)	0.001							
[27]	PENG GA	50.4 (31) 56.4 (31)	0.21								27 (36) 46 (52)	0.02	

Study	Groups	Opioid use intraoperative MME (SD)	*p* **value**	GRADE	First analgesia demand min	*p* **value**	GRADE	Time to discharge min (SD)	*p* **value**	GRADE
**Randomized controlled trials**										
[21]	PENG/LFCN FICB						High			
[24]	PENG FICB				500 165	0.002				
[9]	PENG Sham block									
[1]	PENG Sham block									
**Retrospective comparative cohort studies**										
[6]	PENG QL	40 [30, 60]^a^ 40 [30, 60]^a^	0.6408	Very low			Low			Very low
[19]	PENG/LFCN Neuraxial PENG Neuraxial									
[35]	PENG GA GA/Marcaine									
[38]	PENG GA	16.9 (14.1) 40.6 (18.3)	<0.001							
[20]	PENG GA	10.85 (3.96) 13.72 (5.35)	0.04					81.5 (19) 95.8 (31)	0.05	
[27]	PENG GA	6.6 (2.4) 7.5 (2.3)	0.01		15.8 12.3	0.23		165.0 202.8	0.04	

Abbreviations: BKK, bupivacaine, ketamine and ketorolac; GA, general anaesthesia; GRADE, Grades of Recommendation Assessment, Development and Evaluation; FICB, fascia iliaca compartment block; IQR, interquartile range; LFCN, lateral femoral cutaneous nerve; MME, milligram morphine equivalent; PACU, post anaesthesia care unit; PENG, pericapsular nerve group; PONV, post‐operative nausea and vomiting; QL, quadratus lumborum; SD, standard deviation.

^a^ These indicate median [IQR] scores, not mean scores.

#### Post‐operative motor weakness

One RCT measured quadriceps femoris muscle strength (MMT) at 6, 12, 18, 24, 26 and 48 h post‐operatively, observing higher median muscle strength scores in the PENG/LFCN group (3, 3, 4, 5, 5, 5) compared to the FICB group (1, 2, 2, 3, 4, 4) [21]. Another RCT assessed quadriceps strength using a leg‐lifting test, where all patients in the PENG group successfully raised their operated leg, while only 60% of the FICB control group could do the same [24]. A third RCT reported no incidents of falls or muscle weakness in either group [9]. One retrospective comparative cohort study found no motor nerve palsy in the PENG group, although control group results were not specified [20].

#### Total time spent in the PACU

**Figure 3:**
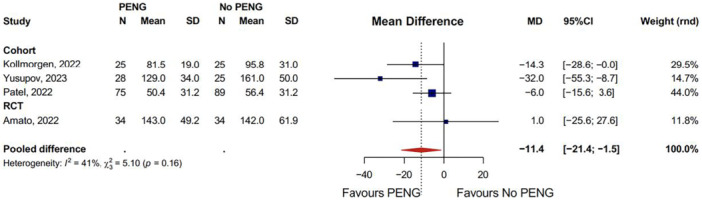
Pooled total time spent in the PACU. CI, confidence interval; MD, mean difference; PACU, post anaesthesia care unit; PENG, pericapsular nerve group; RCT, randomized controlled trial; SD, standard deviation.

Pooled data from three retrospective comparative cohort studies and one RCT confirmed that PENG groups had significantly reduced PACU stays compared to no block or sham block (MD: −11.4; 95% CI: −21.4 to −1.5) (Figure 3) [1, 6, 19, 20, 27, 35, 38].

#### Total opioid consumption in the PACU

**Figure 4:**
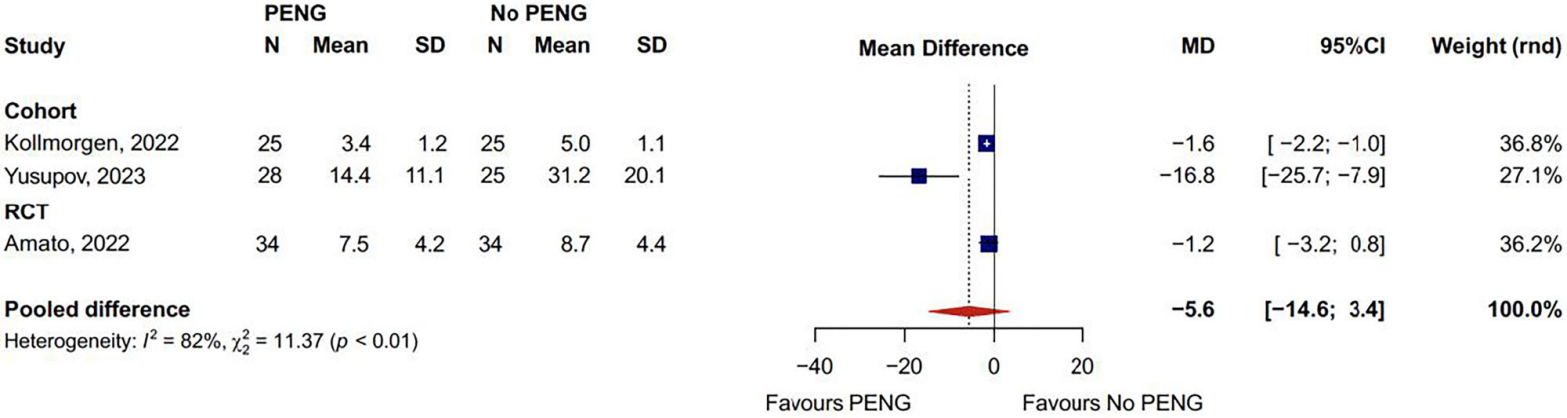
Pooled total opioid consumption in the PACU. CI, confidence interval; MD, mean difference; PACU, post anaesthesia care unit; PENG, pericapsular nerve group; RCT, randomized controlled trial; SD, standard deviation.

Pooled data (Figure 4) revealed a non‐significant reduction in mean PACU opioid consumption with the PENG block compared to sham or no block (MD: −5.6, 95%CI: −14.6 to 3.4) [1, 20, 38]. Additionally, one retrospective comparative cohort study found significantly lower total oral opioid consumption at the PACU in patients who received both PENG and LFCN blocks compared to those with no block, which was however not seen in PENG only [19]. One retrospective cohort study showed no difference in opioid use between QL and PENG [6]. And one other showed a lower use of opioids in PENG/BKK compared to no block.

#### Total opioid use 24 h post‐operative

No significant difference in total opioid consumption within the first 24 h post‐operatively was found between the PENG group and the control group in two RCTs, whereas a third RCT reported significantly lower opioid use in the PENG group, shown in Table 4 [1, 9, 21, 24]. No retrospective comparative cohort studies recorded 24‐h opioid use, although three studies reported significantly lower inpatient opioid consumption in the PENG group [20, 27, 38].

#### Incidence of PONV

Data on the incidence of PONV were documented by three RCTs, which reported no significant differences between the PENG and control groups, shown in Table 4 [1, 9, 21]. Two retrospective comparative cohort studies reported a lower incidence of PONV in the PENG group (Table 4) [27, 38].

#### Opioid use intraoperatively

**Figure 5:**
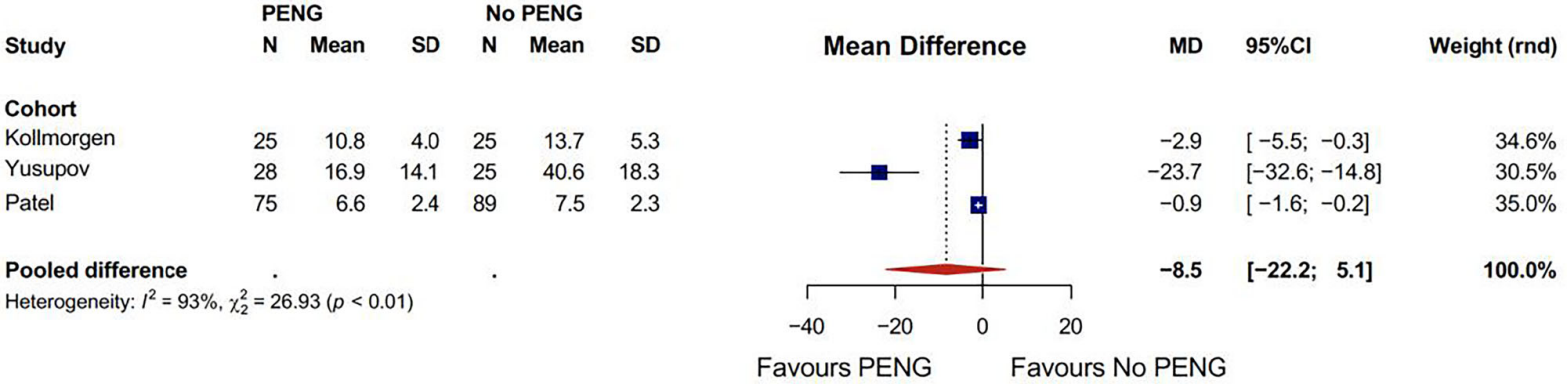
Pooled intraoperative opioid usage. CI, confidence interval; MD, mean difference; PENG, pericapsular nerve group; SD, standard deviation.

Pooled data from three retrospective comparative cohort studies (Figure 5) revealed a non‐significant reduction in intraoperative opioid use with PENG block compared to no block (MD: −8.5, 95% CI: −22.2 to 5.1) [20, 27, 38].

#### First analgesia demand

Two studies, an RCT and a retrospective comparative cohort study, examined first analgesia demand, as shown in Table 4. The RCT revealed a significant prolongation until the first request for analgesia in favour of the PENG intervention, whereas the retrospective comparative cohort study reported no significant difference between the groups (Table 4) [24, 27].

#### Time to discharge

In two retrospective comparative cohort studies, the time to discharge was described. Both documented significantly shorter times in the PENG groups (Table 4) [20, 27].

#### Continued opioid use

One RCT documented outpatient opioid use after 1 week and revealed that more patients in the PENG group than in the control group used opioids (14 vs. 7) [1]. A retrospective comparative cohort study reported that the PENG group consumed fewer opioids (226 MME) than did the control groups (335 and 387 MME) [35].

#### Complications and side effects

All studies reported on complications related to the nerve blocks or analgesia, which were not found in all studies. Operation‐related complications were also not reported.

## DISCUSSION

This systematic review focused on the clinical outcomes of the PENG block in hip arthroscopy. This review of the available literature of both RCTs and retrospective comparative cohort studies found lower maximum and mean VAS scores in the PACU and first 24 h post‐operatively in patients receiving a PENG block. Second, this review also found that patients with PENG spent a shorter time in PACU, had potentially less PONV and less motor weakness, although limited reports. In opioid consumption intraoperatively, at the PACU and post‐operatively, the time to discharge and continued opioid use, no evident differences were found based on the available literature. Caution is advised due to moderate to low GRADE evidence.

Regarding maximum VAS scores in the PACU, one RCT reported significantly lower scores in the PENG group compared to the control group [24]. Similarly, pooled VAS scores from three retrospective comparative cohort studies also revealed lower maximum PACU VAS scores in the PENG groups, although these findings are based on low GRADE evidence [20, 27, 35, 38]. The difference in pain score findings between the RCTs and retrospective comparative cohort studies may reflect variations in surgical procedures and differences in GRADE quality (RCTs moderate, retrospective comparative cohorts low). While some studies have investigated primarily femoroplasty and acetabuloplasty, others have focused on cam repair and debridement. These differences likely influenced the intensity of pain experienced by patients and, consequently, their VAS scores.

The nonsignificant differences in the mean VAS scores reported by Amato et al. and Eppel et al. are supported by the findings of Ellis et al., who examined the effect of a PENG block combined with abdominis plane blocks versus no block in patients undergoing hip arthroscopy and periacetabular osteotomy [1, 8, 9]. They did not find a significant difference in the mean VAS score in the PACU. In contrast, Dusak et al., in a systematic review of nine studies on PENG block in hip fracture surgery and hip arthroplasty, suggested better pain control with PENG than with FN block, FICB or no block [7]. Their findings align with this review, showing slight improvements in the PACU and 24‐h mean VAS scores with PENG. Similarly, Orozco et al. reported positive results in hip arthroscopy but highlighted the need for further research due to limited data [25].

The potential for a placebo effect must be considered. Noaman et al. used FICB for comparison, whereas Amato et al. and Eppel et al. relied solely on a sham block [1, 9, 24]. This methodological difference could have induced a placebo effect, potentially distorting patients' pain experiences in a manner distinct from the effects of FICB. The lack of significant differences in VAS scores reported by Amato et al. and Eppel et al. may further suggest that a placebo effect played a role, given that the RCTs used sham blocks, whereas the retrospective comparative cohort studies had no block in their control groups. This assertion is supported by a study by Shariat et al., which explored the effects of FICB against a sham block, similarly finding no difference in post‐operative pain intensity or opioid consumption between groups [31].

Combining PENG with other blocks or infiltrations may enhance analgesia. For example, Widmeyer et al. combined PENGs with BKK, whereas Coffman et al. and Kim et al. combined PENGs with LFCNs, potentially improving pain control beyond PENGs alone [16, 19, 35]. Kim et al. reported that the PENG‐LFCN group had significantly lower pain scores than the control group, whereas the PENG‐only group did not. Similarly, Morrison et al. reported favourable outcomes with PENG combined with local infiltration or FNB, with minimal opioid use up to 72 h post‐operative [23]. These findings suggest that combined approaches may offer optimal analgesia.

Motor weakness following the PENG block is an important consideration. Morrison et al. reviewed 20 studies involving 74 patients with hip fractures and other hip surgeries and reported only two cases of muscle weakness, both of which were due to incorrect anaesthetic deposition [23]. However, the studies in this review did not sufficiently address quadriceps muscle weakness. Noaman et al. and Liu et al. assessed quadriceps strength and reported no motor nerve palsy in 23 patients receiving PENG and 39 receiving PENG combined with LFCN [2, 13]. In contrast, both control groups, respectively 20 and 39 patients who received FICB, presented more cases of muscle weakness, 40% in the study of Noaman and a lower median strength score in the study of Liu et al. Kollmorgen et al. reported similar results for PENG, although they did not specify control group outcomes, which involved general anaesthesia, raising concerns of publication bias [20]. Eppel et al. reported no muscle weakness or falls in either group [9]. These findings align with those of Giron et al., who emphasized PENG's ability to reduce pain without causing quadriceps weakness and Dusak et al., who noted greater mobility in PENG recipients than FICB or no block [7, 12].

This motor‐sparing characteristic positions PENG as a preferable alternative to other techniques, particularly FICB, which is associated with delayed motor recovery that can hinder early post‐operative mobilization [23]. Other effective techniques, such as FICB, FNB, QL block and PI, have also been shown to reduce pain in studies, with some reporting minimal muscle weakness [3, 22, 28, 29, 30, 34, 36, 37]. However, findings remain mixed, particularly concerning opioid use, discharge time and PACU stay. The ability of the PENG to combine effective analgesia with minimal motor impairment highlights its potential advantages in facilitating early rehabilitation and improving clinical outcomes.

Three RCTs included in this review reported opioid consumption 24 h after surgery [1, 9, 24]. While one RCT reported lower opioid use in the PENG group, the others reported no significant difference. This might be due to the varied doses of analgesics administered in each group. The study that reported significant differences used a higher dose of analgesics than the other studies did, which reported no significant differences between groups. This might have amplified the analgesic effect, thereby reducing opioid consumption more effectively in the PENG group. Additionally, one RCT faced recruitment challenges, with many patients refusing to participate, possibly because they had previously undergone hip arthroscopy and did not want to risk being placed in the sham block group [1]. This may have introduced selection bias, potentially excluding patients with greater pain sensitivity or a greater likelihood of benefiting from the PENG block. Similarly, two retrospective comparative cohort studies listed prior arthroscopy as an exclusion criterion, which may have been for this same reason [20, 27].

## LIMITATIONS

This systematic review included both RCTs and retrospective comparative cohort studies comparing the PENG block to other analgesic methods. However, significant heterogeneity among the studies limits direct comparisons. Variations in analgesic interventions, doses of administered analgesics, surgical indications, pain score measurement tools (NRS vs. VAS) and differences in outcomes such as the mean pain scores, maximum pain scores, pain scores measured at different times, total opioid use, intraoperative opioid use and PACU opioid use make comparisons challenging. Another limitation is the lower quality of evidence in retrospective comparative cohort studies, with several outcomes rated low to very low GRADE ratings for certain outcomes. These issues include insufficient or unreported blinding, inadequate or poorly described randomization and unclear group comparability. Additionally, the potential for biases, including publication bias, weakens the reliability of these findings.

## STRENGTHS

To present our findings on this subject in the most transparent and objective way, we followed the PRISMA guidelines for systematic reviews. We performed a qualitative and quantitative analysis, examining any potential bias and qualifying the strength of evidence via the Cochrane checklists and the GRADE method, thereby providing a comprehensive overview of studies exploring PENG block in hip arthroscopy within a single review.

## RECOMMENDATIONS

The findings of this systematic review are encouraging and highlight the potential of the PENG block in hip arthroscopy. This technique is associated with minimal to no complications, making it a safe option for patients. This review underscores the established safety, effectiveness, and potential superiority of PENG blocks. However, further RCTs with larger sample sizes are necessary to better evaluate its effectiveness and to further investigate its impact on motor function. Given the positive results thus far and the potential to avoid muscle weakness, a logical next step would be a well‐designed RCT to directly compare PENG, QL and PI blocks. This would provide valuable insights into their relative effectiveness and clinical utility. Additionally, it is essential to compare the PENG block to a broader range of existing practices in studies where outcomes are measured in a homogeneous manner and described at the same time points. This approach will help determine its full utility in clinical practice.

## CONCLUSION

The current literature suggests that PENG block offers potential for effective post‐operative pain relief, decreased opioid consumption 24 h after surgery and minimally decreased PONV without motor function impairment, yet its impact on hospital stay and PACU opioid use appears minimal. Although the PENG block could still be a viable option for hip arthroscopy, given its minimal risks, the outcomes of this review should be considered preliminary, due to moderate to low GRADE evidence, highlighting the need for further research.

## AUTHOR CONTRIBUTIONS

All authors contributed to the study conception and design, especially Daniel Haverkamp as the hip arthroscopy specialist. Jeroen den Breejen, Justin van Loon and Michel H. Klaver were involved in the screening and selection process. Data collection was performed by Jeroen den Breejen, Justin van Loon and Inger N. Sierevelt. Inger N. Sierevelt conducted the statistical analysis. The first draft of the manuscript was written by Jeroen den Breejen, together with Justin van Loon. All authors commented on previous versions of the manuscript. All authors read and approved the final manuscript.

## CONFLICT OF INTEREST STATEMENT

The authors declare no conflicts of interest.

## ETHICS STATEMENT

No ethical approval or informed consent was needed due to the systematic review design of this study.

## Data Availability

These data were derived from the following resources available in the public domain: PubMed (https://pubmed.ncbi.nlm.nih.gov/), Embase (https://www.embase.com/) and the Cochrane Library (https://www.cochranelibrary.com/).

## APPENDIX A SEARCH STRATEGY

**PubMed**

(‘Nerve Block’[MeSH] OR nerve block*[tiab] OR peripheral block*[tiab]) OR ((pericapsular nerve group*[tiab] OR PENG[tiab] OR nerve group*[tiab]) AND block*[tiab]) AND (‘Arthroscopy’[Mesh] OR arthroscop*[tiab]) AND (‘Hip’[Mesh] OR hip[tiab] OR hips[tiab] OR coxa[tiab] OR coxas[tiab])

**Embase (Ovid)**:

**Table:**

#	Search	Results
1	exp nerve block/	50,913
2	(nerve block* or peripheral block*).ti,ab,kf.	23,102
3	((pericapsular nerve group* or PENG or nerve group*) and block*).ti,ab,kf.	353
4	1 or 2 or 3	55,187
5	exp arthroscopy/	40,103
6	arthroscop*.ti,ab,kf.	49,922
7	5 or 6	59,764
8	exp hip/	122,920
9	(hip or hips or coxa or coxas).ti,ab,kf.	231,563
10	8 or 9	289,483
11	4 and 7 and 10	196

**Cochrane Library**

**Table:**

ID	Search	Hits
#1	(nerve block* or peripheral block*):ti,ab,kw	20,197
#2	((pericapsular nerve group* or PENG or nerve group*) and block*):ti,ab,kw	11,939
#3	#1 or #2	20,223
#4	(arthroscop*):ti,ab,kw	5970
#5	(hip or hips or coxa or coxas):ti,ab,kw	29,937
#6	#3 and #4 and #5 in Cochrane Reviews, Trials	97

## APPENDIX B RISK OF BIAS COCHRANE COLLABORATION's TOOL FOR RCTs

**Table:**

Study	Selection bias	Selection bias	Performance bias	Detection bias	Attrition bias	Reporting bias	Other sources of bias
	Random sequence generation	Allocation concealment	Blinding of participants	Blinding of primary outcome assessors	Incomplete primary outcome data	Selective reporting	Funding, baseline characteristics of trial arms
[21]							
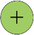
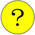
[24]							
[1]							
[9]							

## APPENDIX C

See Table C1.

**Table C1:** Risk of bias assessment using methodological items for non‐randomized studies score (MINORS), summary of the included cohort and randomized controlled trials.

	MINORS^b^												
Study	1	2	3	4	5	6	7	8	9	10	11	12	Total^a^
**RCT**													
[21]	2	2	2	2	2	2	2	1	2	2	2	2	**23**
[24]	2	2	2	2	2	2	2	2	2	2	2	2	**24**
[1]	2	2	2	2	2	2	2	2	2	2	2	2	**24**
[9]	2	2	2	2	2	2	2	2	2	2	2	2	**24**
**Non‐randomized comparative studies**													
[6]	2	1	1	2	0	2	2	0	0	2	2	1	**15**
[19]	2	2	1	2	0	2	2	1	2	2	2	2	**20**
[35]	2	2	1	2	0	2	2	0	2	1	1	2	**17**
[38]	2	2	1	2	0	2	2	0	2	2	2	2	**19**
[20]	2	1	1	2	0	2	2	0	2	2	2	2	**18**
[27]	2	2	1	2	0	2	2	0	2	2	1	2	**18**

^a^ The global ideal score is 16 for non‐comparative studies and 24 for comparative studies.

^b^ The items are scored 0 (not reported), 1 (reported but inadequate) or 2 (reported and adequate).

1. A clearly stated aim: the question addressed should be precise and relevant in the light of available literature.

2. Inclusion of consecutive patients: all patients potentially fit for inclusion (satisfying the criteria for inclusion) have been included in the study during the study period (no exclusion or details about the reasons for exclusion).

3. Prospective collection of data: data were collected according to a protocol established before the beginning of the study.

4. End points appropriate to the aim of the study: unambiguous explanation of the criteria used to evaluate the main outcome, which should be in accordance with the question addressed by the study. Also, the end points should be assessed on an intention‐to‐treat basis.

5. Unbiased assessment of the study end point: blind evaluation of objective end points and double‐blind evaluation of subjective end points. Otherwise, the reasons for not blinding should be stated.

6. Follow‐up period appropriate to the aim of the study: the follow‐up should be sufficiently long to allow the assessment of the main end point and possible adverse events.

7. Loss to follow up less than 5%: all patients should be included in the follow up. Otherwise, the proportion lost to follow up should not exceed the proportion experiencing the major end point.

8. Prospective calculation of the study size: information on the size of detectable difference of interest with a calculation of 95% confidence interval, according to the expected incidence of the outcome event, and information about the level for statistical significance and estimates of power when comparing the outcomes.

9. An adequate control group: having a gold standard diagnostic test or therapeutic intervention recognized as the optimal intervention according to the available published data

10. Contemporary groups: control and studied group should be managed during the same time period (no historical comparison).

11. Baseline equivalence of groups: the groups should be similar regarding the criteria other than the studied end points. Absence of confounding factors that could bias the interpretation of the results.

12. Adequate statistical analyses: whether the statistics were in accordance with the type of study, with calculation of confidence intervals or relative risk.

## APPENDIX D

See Table D1.

**Table D1:** GRADE assessment.

Certainty assessment							No. of patients		Effect			
No. of studies	Study design	Risk of bias	Inconsistency	Indirectness	Imprecision	Other considerations	PENG block	Currently available practices	Relative (95% CI)	Absolute (95% CI)	Certainty	Importance
**Mean VAS PACU (Scale from: 0 to 10)**												
3	Randomized trials	Serious^a^^,^ b	Not serious	Not serious	Not serious	None	91	88	—	Not pooled	⊕⊕⊕◯ Moderate^a^^,^ b	CRITICAL
**Mean VAS PACU (Scale from: 0 to 10)**												
3	Non‐randomized studies	Very serious	Not serious	Not serious	Not serious	None	207	233	—	Not pooled	⊕◯◯◯ Very low	CRITICAL
**Max VAS PACU (Scale from: 0 to 10)**												
1	Randomized trials	Not serious	Not serious	Not serious	Not serious	None	23	20	—	**0** (0 to 0)	⊕⊕⊕⊕ High	CRITICAL
**Max VAS PACU (Scale from: 0 to 10)**												
5	Non‐randomized studies	Very serious	Not serious	Not serious	Not serious	None	290	301	—	MD **1.1 lower** (1.7 lower to 0.6 lower)	⊕◯◯◯ Very low	CRITICAL
**Mean VAS 24 h**												
4	Randomized trials	Serious	Not serious	Not serious	Not serious	None^b^	130	127	—	Not pooled	⊕⊕⊕◯ Moderate^b^	CRITICAL
**Muscle weakness**												
3	Randomized trials	Serious^b^	Not serious	Not serious	Not serious	None	96	93	—	Not pooled	⊕⊕⊕◯ Moderate^b^	CRITICAL
**Muscle weakness**												
1	Non‐randomized studies	Very serious	Not serious	Not serious	Not serious	None	25	25	—	**0** (0 to 0)	⊕◯◯◯ Very low	CRITICAL
**Total opioid use PACU**												
5	Non‐randomized studies	Very serious	Not serious	Not serious	Not serious	None	235	244	—	MD **5.6 lower** (14.6 lower to 3.4 higher)	⊕◯◯◯ Very low	IMPORTANT
**Total opioid use 24 h**												
3	Randomized trials	Serious^a^,^b^	Serious^c^	Not serious	Not serious	None^b^	91	88	—	see comment	⊕⊕◯◯ Low^a^^,^ ^b^ ^,^ c	IMPORTANT
**PACU time**												
1	Randomized trials	Serious^a^	Not serious	Not serious	Not serious	None	34	34	—	**0** (0 to 0)	⊕⊕⊕◯ Moderate^a^	IMPORTANT
**PACU time**												
6	Non‐randomized studies	Very serious	Not serious	Not serious	Not serious	None	310	333	—	MD **11.4 lower** (21.4 lower to 1.5 lower)	⊕◯◯◯ Very low	IMPORTANT
**PONV**												
3	Randomized trials	Serious^a^^,^ b	Not serious	Not serious	Not serious	None	107/214 (50.0%)	107/214 (50.0%)	Not pooled	See comment	⊕⊕⊕◯ Moderate^a^^,^ b	IMPORTANT
**PONV**												
3	Non‐randomized studies	Very serious	Not serious	Not serious	Not serious	None	215/441 (48.8%)	226/441 (51.2%)	Not pooled	Not pooled	⊕◯◯◯ Very low	IMPORTANT
**First analgesia demand**												
1	Randomized trials	Not serious	Not serious	Not serious	Not serious	None	23	20	—	**0** (0 to 0)	⊕⊕⊕⊕ High	IMPORTANT
**First analgesia demand**												
1	Non‐randomized studies	Serious	Not serious	Not serious	Not serious	None	75	89	—	**0** (0 to 0)	⊕◯◯◯ Very low	IMPORTANT
**Time to discharge**												
2	Non‐randomized studies	Very serious	Not serious	Not serious	Not serious	None	100	114	—	Not pooled	⊕◯◯◯ Very low	NOT IMPORTANT

Abbreviations: CI, confidence interval; GRADE, Grades of Recommendation Assessment, Development and Evaluation; MD, mean difference; PACU, post anaesthesia care unit; PENG, pericapsular nerve group; VAS, visual analogue scale.

^a^ It was unclear whether outcome assessors were blinded.

^b^ Possible publication bias.

^c^ Outcomes were inconsistent between studies.
